# Contrast-induced acute kidney injury and its contemporary prevention

**DOI:** 10.3389/fcvm.2022.1073072

**Published:** 2022-12-06

**Authors:** Marek Sůva, Petr Kala, Martin Poloczek, Jan Kaňovský, Roman Štípal, Martin Radvan, Jiří Hlasensky, Martin Hudec, Vojtěch Brázdil, Jitka Řehořová

**Affiliations:** ^1^Department of Internal Medicine and Cardiology, University Hospital, Brno, Czechia; ^2^Department of Internal Medicine and Cardiology, Faculty of Medicine, Masaryk University, Brno, Czechia; ^3^Department of Internal Medicine and Gastroenterology, University Hospital, Brno, Czechia

**Keywords:** PCI, nephrotoxicity, acute kidney injury, contrast-induced acute kidney injury, chronic renal insufficiency, prevention

## Abstract

The complexity and application range of interventional and diagnostic procedures using contrast media (CM) have recently increased. This allows more patients to undergo procedures that involve CM administration. However, the intrinsic CM toxicity leads to the risk of contrast-induced acute kidney injury (CI-AKI). At present, effective therapy of CI-AKI is rather limited. Effective prevention of CI-AKI therefore becomes crucially important. This review presents an in-depth discussion of CI-AKI incidence, pathogenesis, risk prediction, current preventive strategies, and novel treatment possibilities. The review also discusses the difference between CI-AKI incidence following intraarterial and intravenous CM administration. Factors contributing to the development of CI-AKI are considered in conjunction with the mechanism of acute kidney damage. The need for ultimate risk estimation and the prediction of CI-AKI is stressed. Possibilities of CI-AKI prevention is evaluated within the spectrum of existing preventive measures aimed at reducing kidney injury. In particular, the review discusses intravenous hydration regimes and pre-treatment with statins and N-acetylcysteine. The review further focuses on emerging alternative imaging technologies, alternative intravascular diagnostic and interventional procedures, and new methods for intravenous hydration guidance; it discusses the applicability of those techniques in complex procedures and their feasibility in current practise. We put emphasis on contemporary interventional cardiology imaging methods, with a brief discussion of CI-AKI in non-vascular and non-cardiologic imaging and interventional studies.

## Introduction

The first report of contrast-induced acute kidney injury (CI-AKI) appeared in 1962 (1). This observation-initiated research of the toxicity and potential harmful effects of contrate media (CM). Because of numerous reports of CI-AKI, imaging procedures involving CM are occasionally withheld (2). However, CI-AKI risk might be considerably lower than initially estimated (3), although CI-AKI is one of the significant and potentially catastrophic complications in patients undergoing procedures with iodinated CM. CI-AKI is also a leading cause of acute renal failure in hospitalized patients, ranking third after ischemic and drug-induced injury (4). The increasing number of interventional and diagnostic procedures using CM calls for an accurate estimate of CI-AKI incidence. Despite current advancements, the treatment of CI-AKI remains relatively limited and mainly focuses on non-dialytic treatment such as avoidance or discontinuation of nephrotoxic drugs, adjustments of hemodynamic parameters and search for reversible causes (5). For all these reasons, the development of proper treatment and prevention methods is vital. As discussed subsequently in more detail, CI-AKI is defined as a decrease in renal functions up to 3 days after the CM administration (6). The severity of consequent acute kidney injury may range from a mild transient serum creatinine elevation to a severe renal failure requiring renal replacement therapy. The pathogenesis of CI-AKI is still not fully understood, although the current research of CI-AKI allows targeting specific mechanisms. The understanding of these mechanisms appears to allow both early diagnosis and prevention. As the treatment of CI-AKI is relatively complex, the prevention and early detection are of substantial importance.

To summarize the present understanding of CI-AKI, this review discusses the contemporary methods for CI-AKI prevention and for its risk estimation. The incidence of CI-AKI and the discrepancies in the relevant reports are described together with their implications for prevention strategies. The review concentrates primarily on imaging techniques of interventional cardiology and discusses CI-AKI mitigating methods including device-guided hydration, novel contrast-free imaging, and the emerging pharmacologic pre-treatment possibilities. The review also presents suggestions for further research needed to obtain an evidence-based assessment of the effectiveness and feasibility of novel approaches to CI-AKI.

## Methods

PubMed, Cochrane Library database, and Embase have been searched for publications of Clinical Trials, Meta-analyses, Randomized Controlled Trials, Reviews, and Systematic Reviews with the following keywords: ACUTE AND (“KIDNEY INJURY” OR NEPHROPATHY OR “KIDNEY DISEASE” OR “RENAL FAILURE”) AND “CONTRAST MEDIA”. Publications in English were considered if referring to an adult population (>18 years of age). These searches found 360 articles up to the publication year of 2021. Subsequently, the publications were screened to exclude duplicates and editorial comments.

## Definition

The definition of acute kidney injury (AKI) and of CI-AKI varies in different publications (7–9). Indeed, systematic review published by NICE showed limited agreement among definitions concerning AKI detection and severe AKI outcome prediction (10). In this review, we used the AKI definition by the Kidney Disease Improving Global Outcomes working group (11). This definition has also been used in the most recent guidelines by French nephrology societies and the French radiological society, as published in 2021 (6). According to this definition, either the following criteria define CI-AKI if occurring within 48–72 h after CM administration: (a) an increase in serum creatinine of ≥26.4 μmol/L (0.3mg/dl); (b) an increase in serum creatinine ≥ 1.5–1.9 times from baseline; (c) decrease in the urine output <0.5 ml/kg/h. Importantly, CI-AKI needs to be differentiated from post-contrast acute kidney injury without suspected causality between CM administration and AKI. Thus, CI-AKI needs to be considered when no CM-unrelated etiology of AKI is plausible. Although CI-AKI often presents as only transient kidney injury, it may occasionally progress to the significant stage of renal failure that worsens clinical outcome and significantly increases hospital expenditure. When considering and diagnosing CI-AKI, minor changes in serum creatinine have to be interpreted with caution, as they may be affected by fluid shifts, medication administration, and diurnal rhythms (12).

## Incidence

**FIGURE 1:**
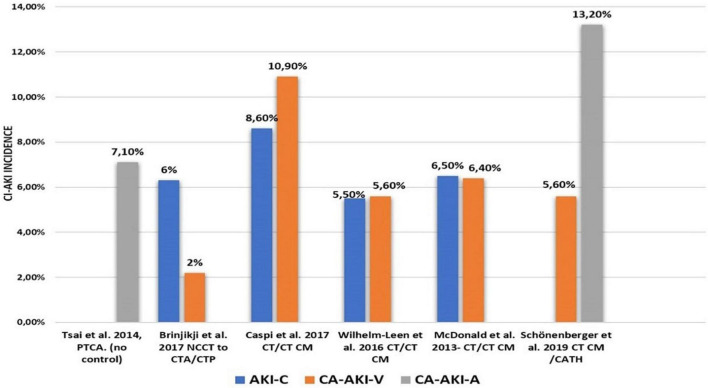
Incidence of Cl-AKI reported in selected recent studies. Tsai et al. (21); McDonald et al. (23); Wilhelm-Leen et al. (24); Schönenberger et al. (27); Ward et al. (29); Kiss and Hamar (30). CI-AKI, contrast induced acute kidney injury; PTCA, percutaneous transluminal coronary angioplasty; CTA, computed tomography angiography; CTP, computed tomography perfusion; NCCT, noncontrast computed tomography; CTCM, compute tomography with contrast; CATH, coronary vessels catheterisation; AKI-C, acute kidney injury control; CA-AKI-V, venous contrast application as the causative factor of CA-AKI, CA-AKI-A, arteria contrast application as the causative factor of CA-AKI.

The true CI-AKI incidence has recently been discussed (13–15). Previously published estimates suffered from the differences in confounding factors, varying study designs and observational nature of studies. Initial descriptive studies were also mostly uncontrolled. While the initial risk of CI-AKI has been grossly overestimated with a reported incidence of up to 20–30% (16, 17) contemporary clinical data analysis has shown it to be much less common (18, 19). Studies comprising control groups not exposed to CM (20), report the CI-AKI incidence of approximately 2–6% (21) (Figure 1). This incidence number is almost tenfold lower than initially estimated. As more recent studies show lower CI-AKI incidence than older reports, it remains speculative whether such a decrease has been caused by improved statistical methodologies, effective prevention protocols, or lower CM doses.

Interestingly, some studies found no increase in CI-AKI incidence after CM administration in comparison to a clinically matched control population who received no CM (13, 22). Meta-analysis by McDonald et al. found no impact of CM administration on CI-AKI incidence in patients undergoing computed tomography investigations (23). Wilhelm-Leen et al. published similar results based on the so-far largest retrospective analysis (data of up to six million patients interpreted) (24). These findings were further supported by studies reporting a high incidence of daily creatinine fluctuations (12). Such transient fluctuations might contribute to CI-AKI diagnosis if based on minor plasma creatinine increments.

The role of CM in nephropathy is possibly occasionally overestimated. Nevertheless, the risk of CI-AKI is not negligible. Recent propensity score-matching meta-analysis by Gorelik et al. showed significantly increased likelihood of CI-AKI patients with advanced renal insufficiency who underwent radiocontrast-enhanced computed tomography (25). Similar higher incidence of CI-AKI was shown in a retrospective study by Aubry et al. who reviewed patients undergoing cardiovascular procedures (26). Schönenberger E et al. and McCullough et al. reported profound risk of CI-AKI in patients with advanced chronic kidney disease after receiving high CM doses intraarterially (27, 28). Recent meta-analysis by Song et al. showed that elderly patients have at least a twofold increase in the CI-AKI risk (18). These findings could possibly lead to lesser enrollment of elderly patients with kidney disease into imaging studies and form a unintentional bias known as “renalism” (2).

Hence there are number of studies that dispute the absence of CI-AKI risk, especially if considering elderly patients or those with advanced chronic kidney disease. Adequately powered and well controlled clinical registry is required to determine the actual risk of CI AKI under different conditions.

## Pathogenesis

The pathogenesis of CI-AKI is still not fully understood. Nevertheless, a consensus exists that it is caused by a combination of direct renal toxicity by CM and of the hemodynamic perturbations due to CM administration that led to indirect renal toxicity.

### Direct mechanisms

The direct toxicity of CM was observed and studied both *in vitro* using isolated or cultured human tubular cells, and *in vivo* using animal models. While all CM types are cytotoxic *in vitro*, the detailed cytotoxicity mechanisms can only be approximated. Cellular disarray is observed but the details of physiologic pathways that lead to cellular necrosis remain unknown. Translation to clinical practice and clinical studies is rather problematic. This may be, in part, due to the concentrations of CM used in animal studies (usually over 5–100 times the concentrations found in plasma of patient undergoing CM administration). As proposed by Ward et al. (29) changes of cellular homeostasis and cellular disarray induced in animal studies would not occur at clinical practice. The chemical structure of CM may suggest that free iodine particles lead to cytotoxicity. *In vitro* studies suggest that the intravascular injection of CM may cause fenestration of endothelial layer, vacuolization of cells, and eventually cellular apoptosis (30). Nevertheless, administration of CM alone rarely causes apparent destruction of renal cells in animal models, unless additional insult is present (30). CM-induced cellular disarray has been associated with the induction of pro-apoptotic pathways and to the decreased activation of kinases needed for cell survival (31). These mechanisms might be more pronounced in renal cells, primarily because of the tubular CM concentration which exposes tubular cells to increased direct damage (32). The consequence of this cellular injury leads indirectly to further kidney injury. More specifically, apoptotic and/or necrotic cells shed to renal tubules and might ultimately lead to renal obstruction.

Clinically relevant doses of CM cause cytotoxicity via a complex process. Recent *in vitro* models using human kidney cells (immortalized, not derived from malignant cells, with viable ion organic transporters capable of up taking CM) revealed that mitochondrial viability decreased considerably faster than cell viability. As a result, it is hypothesized that mitochondrial cells, as one of the first structures in cells, are damaged. In clinically relevant CM exposure, the previously suggested mechanism of misfolded or unfolded protein accumulation with subsequent endoplasmic reticulum stress does not play a critical role in decreasing cell viability (29). One of the main mechanisms of cellular death is a calcium overload within mitochondria. The pathway of cell death begins with mitochondrial membrane permeability transitions and starts with the accumulation of calcium within mitochondria. Apoptosome activation is occurring as the mitochondrial membrane becomes more permeable to larger solutes and proteins. This eventually leads to apoptosis. The exact source of calcium is yet unknown, but due to the partial action of external and intracellular calcium chelators, it is most likely the combination of both. Calcium inflow also activates calpains (calcium activated proteases). Calpains are required for the activation of certain pro-apoptotic caspases involved in the pathophysiology of CI-AKI. This was demonstrated by the pre-treatment with a calpain inhibitor, which reduced the incidence of CI-AKI in rats (33).

### Indirect mechanisms

The unique architecture of renal tubules enables the physiological function of the outer medulla even under low oxygen saturation (34). The limited regional oxygen supply and high local oxygen need for tubular reabsorption make outer medulla particularly susceptible to minute renal flow and oxygen saturation changes. CM cannot be tubularly absorbed, making it responsible for potent tubular congestion (35). Most of the tubularly filtered fluids get reabsorbed which leaves highly concentrated and viscous CM in the remaining fluid. Tubular congestion is further enhanced by iso-osmolar CM since osmolality reduction increases viscosity considerably (36). The slower flow of glomerular fluid leads to pressure build-up which ultimately decreases glomerular filtration (37). CM presence in renal and systemic circulation leads to transient vasodilation of renal vasculature, followed by profound vasoconstriction of vasa recta mediated by the release of vasoactive substances, such as the endothelins, adenosine, intracellular Ca2+, and to the uptake of nitric oxide, a potent vasodilator, through the generation of reactive oxygen substances (38, 39). Above-mentioned principles led to the development of theophylline therapy as a potential targeted therapy to antagonize effect of adenosine (40). Similarly, prostaglandin E1, aloprostadil and inorganic nitrate were examined as potential alleviator of CI-AKI incidence (41, 42). Prolonged vasoconstrictions cause decreased oxygen supply and eventually damage outer medulla. These pathways lead to a decrease in urine flow rate and further stasis of CM in the tubules. Consequently, this enhances the potential of direct CM cytotoxicity due to the prolonged exposure of tubular cells to CM. This effect is intensified especially in the relatively hypoxic renal medulla, which is more susceptible to hypoxemic damage (43).

Ongoing hypoxic injury in the highly metabolic active renal cell causes uncontrolled production of reactive oxygen species (ROS). This eventually overwhelms cells innate antioxidant systems and damages cells via interaction with intracellular organelles and proteins (44). Exposure to CM induces oxidative stress. This leads to the accumulation of proteins with carbonylated side chains which are highly indicative of increased oxidative stress. However, the production of such products is relatively late and therefore could be due to the damages that are already caused by another mechanisms (29). The generation of ROS in mitochondria raises dramatically under conditions of cellular disarray and metabolic disturbances associated with CI-AKI. This ultimately leads to ROS interactions with the mitochondrial membrane and leakage of cytochrome c which then ultimately forms into apoptosome (45). ROS also upregulate signal pathways that activate intrinsic apoptotic pathways (46). These studies formed the basis for the use of antioxidants such as Ascorbic Acid (vit. C), N-acetylcysteine, and Alpha-Tocopherol (47). The level of oxidative stress can be indirectly measured by the reduction in thiol levels as they get consumed by peroxidase. Similarly, we can measure an increase in urinary peroxides and urinary thiobarbituric acid reactive substances as the by-products of lipid peroxidation which can be measured in the early stages of CI-AKI (48). This further proves that the kidney is in hyperoxidative state when exposed to CM.

## Risk stratification and prediction

Presently, the treatment of CI-AKI may still be rather limited. Only supportive measures, such as hemodynamic parameters adjustment or avoidance of nephrotoxic medication, might be considered (5). A certain number of CI-AKI patients eventually progress to end-stage renal disease. Therefore, risk stratification is needed to prevent CI-AKI in patients CM-based undergoing the application of CM.

Altogether, at least 22 stratification models predicting the risk of CI-AKI in patients undergoing percutaneous coronary intervention (PCI) have been developed. As depicted in Table 2 Tsai et al. and Gurm et al. stratification models have the largest derivation and validation population as well as multicentric nature of trial. Both stratification models also include only preprocedural characteristic, making them more useful and easier to use in clinical practice. Notably, only some stratification models have been independently externally validated. Subsequent trials that verified certain stratification models are depicted in Table 1.

**TABLE 1:** External validation and comparative studies of risk stratification models.

Name	Number of prediction models validated	Robustness of study (No. of patients)	Population	Highest C value with 95% CL
Liu et al. (169)	6	Single center (422)	Undergoing PCI for STEMI	Gao et al. 0.876 (0.78–0.97) for CIN Narrow
Ma et al. (170)	7	Single center (7.888)	Undergoing PCI	Brown et al. 0.62 (0.60–0.65) for CIN Broad
Yin et al. (54)	8	Single province (9.013)	Undergoing CAG or PCI	Chen et al. 0.76 (0.63–0.89) for CIN Narrow
Serif et al. (51)	17	Single center (1.247)	Undergoing PCI	Maioli et al. 0.735 for CIN Narrow

CIN Narrow, increase in serum creatinine levels of ≥0.5 mg/dl (44.2 μmol/L) mg/dl; CIN Broad, increase in serum creatinine levels of ≥0.5 mg/dl (44.2 μmol/L) mg/dl or a an 25% increase in baseline Serum creatinine.

**TABLE 2:** Risk stratification models.

Name of study	No. of patients(D/V)	C statistic D/V (original paper)	Characteristics	Only preprocedural	Population	No. of risk factors	Model variables
Bartholomew et al. (171)	20.479 (10 481/9998)	0.89 (missing 95% CL)	Single center	No	Undergoing PCI	8	eGFR <60 ml/min, IABP, urgent/emergent procedure, diabetes, heart failure, hypertension, peripheral artery disease, contrast volume >260 ml
Marenzi et al. (172)	1.172 (218/891)	0.60 (missing 95% CL)	Single center	No	PCI for STEMI	5	Age >75 years, anterior infarction, time to reperfusion, contrast volume, use of IABP
Mehran et al. (49)	8.357 (5571/2786)	0.67 (95% CI 0.608–0.779)	Single center	No	PCI for STEMI	8	Hypotension, heart failure, CKD, diabetes, age >75 years, anemia, use of IABP, contrast volume
Ghani and Tohamy (173)	347 (247/100)	0.61 (missing 95%CL)	Single center	No	Undergoing PCI	5	Basal serum creatinine >115 μmol/L, shock, female gender, multivessel PCI, and diabetes
Maioli et al. (174)	1.783 (1281/502)	0.82 (missing 95%CL)	Single center	Yes	Undergoing CAG or PCI	7	Age >73, diabetes, LVEF 45%, creatinine 1.5 mg/dl or CrCl eGFR ≤ 45(ml/min/1.73 m^2^) ml/min, post hydration creatinine
Fu et al. (175)	895 (668/277)	0.79 (missing 95%CL)	Single center	No	Undergoing PCI	9	eGFR <60 ml/min/1.73 m, diabetes, LVEF < 45%, MI, emergency PCI, myocardial infarction anemia, contrast volume >200 ml, hypotension, age > 70 years
Tziakas et al. (176)	688 (488/200)	0.76 (95%CI 0.685–0.833)	Single center	No	Undergoing PCI	5	CKD, metformin use, prior PCI, peripheral arterial disease, contrast volume ≥ 300 ml
Gurm et al.(52)	60.573 (40,001/20,572)	0.84 (95% CI: 0.821 to 0.857)	Multi center	Yes	Undergoing PCI	15 (reduced model)	PCI indication/status, CAD presentation, cardiogenic shock, heart failure, prePCI LVEF, diabetes, age, weight, height, CK-MB, creatinine, hemoglobin, troponin I, and troponin T
Chen et al. (55)	2.500 (1500/1000)	0.82 (95% CL: 0.78-0.86)	Single center	Yes	Undergoing PCI	9	Age ≥ 70, prior MI, diabetes, hypotension, LVEF ≤ 45%, anemia, eGFR ≤ 45(ml/min/1.73 m^2^), HDL < 1 mmol/, Urgent PCI
Gao et al. (177)	3.945(2.764/1181)	0.71 (95% CI 0.63–0.79)	Singe center	No	Undergoing CAG or PCI	7	Age > 60, hypertension, acute MI, heart failure, IABP usage, CKD, contrast volume > 100 ml
Liu et al. (178)	1.272 (848/424)	0.76 (95% CI 0.685–0.832)	Single center	Yes	Undergoing CAG or PCI	4	IABP, eGFR, Hypoalbuminemia, age
Victor et al. (179)	1.200 (900/300)	0.95 (Spec. 82%, Sens. 92%)	Single center	No	Undergoing PCI	7	GFR value, amount of contrast, hemoglobin, diabetic microangiopathy, hypotension, albuminuria, peripheral vascular disease
Duan et al. (180)	1.777 (1076/701)	0.8 (95%CI: 0.727-0.870)	Single center	Yes	Undergoing CAG or PCI	7	Age ≤ 70, history of myocardial infarction, diabetes, hypotension, LVEF < 45%, anaemia, eGFR
Caspi et al. (181)	1.200 (900/300)	0.81(95% CL 0.79-0.83)	Single center	No	Undergoing PCI	7	independent predictors of AKI age ≥ 70 years, DM insulin, diuretics, anterior MI, eGFR value, Killip III-IV, IABG, LVEF ≤ 45%
Brown et al. (182)	115.633 (87 728/27 905)	0.74 (95% CI: 0.74–0.75)	Multi center	No	Undergoing CAG or PCI	17	Diabetes, peripheral vascular disease, prior stroke, CHF, urgency status, shock, ACS, anemia, low albumin, prior AKI, CKD, prior MI, prior CABG, smoker, age, hypertension, thrombolytic use
Lian et al. (183)	1.286(756/530)	0.7 (95% CL 0,624-0,765)	Single center	Yes	Undergoing CAG	3	age > 75 years, CHF, CrCl < 60 ml/min
Inohara et al. (184)	5.936 (3957/1979)	0.8 (95% CL 0.783-0.815)	Multi center	Yes	Undergoing PCI	7	Age, NYHA III-IV, DM, prior PCI, hypertension, CKD, ACS
Lin et al. (185)	692 (461/231)	0.83 (95% CL 0.776-0.853)	Single center	No	Undergoing emergent PCI	4	Hypotension, Age>75 years, IABP, SCr>1.5 mg/dl
Yuan et al. (186)	1.061 (761/300)	0.76 (95% CI: 0.672-0.841)	Single center	Yes	Undergoing emergent PCI	5	BSA < 1.60 m^2^, history of TIA/stroke, WBC 15.0 × 109/L, eGFR 60 ml/min/1.73 m^2^, IABP usage, diuretics usage
Tsai et al. (187)	947.012 (662,504/284,508)	0.71 (95% CL 0.701, 0.716)	Multi center	Yes	Undergoing PCI	11	Age, prior 2 weeks HF, GFR, diabetes, prior HF/CVD, NSTEMI/UA, STEMI, prior card shock or cardiac arrest, anemia, IABP
Freeman et al. (188)	16.592 (10,729/5863)	0.89 (missing 95%CL)	Multi center	Yes	Undergoing PCI	5	Peripheral artery disease, diabetes, CKD (Cr > 2 mg/dl), heart failure, cardiogenic shock

D, derivation population, V, validation population; IABP, intraaortic balloon pump; CrCl, creatinine clearance; LVEF, left ventricular ejection fraction; CAD, coronary artery disease; PCI, percutaneous coronary intervention; CKD, chronic kidney disease; BSA, body surface area; ACS, acute coronary syndrome; CHF, chronic heart failure; UA, unstable angina pectoris; STEMI, ST-elevation myocardial infarction; NSTEMI, non-ST-elevation myocardial infarction.

In 2004, Mehran et al. (49) proposed one the first objective risk score for CI-AKI risk combining eight variables: systolic blood pressure < 80 mm Hg, intra-aortic balloon pump, NYHA class III/IV, age >75 years, anemia defined by baseline hematocrit value <39% for men and <36% for women, diabetes mellitus, CM volume (1 point for every 100 ml) and estimated glomerular filtration rate less than 60 ml/min/1.73 m^2^ (more points for more severe reduction of kidney function). This risk score is presently used in many catheterization laboratories and has been repeatedly externally validated (50, 51). The risk score by Mehran et al. involves some parameters that can be obtained only during the procedure and make the clinical application of the score challenging.

Subsequent to the proposal by Mehran et al. (49) the development of a sufficiently accurate and easy-to-use prognostic model was attempted, stressing the importance of using only preprocedural factors, thus making the assessment of risk available before the procedure. The risk score published by Gurm et al. (52) utilizes 15 clinical and laboratory indices, usually known before the procedure. Although this risk score applies only to percutaneous coronary interventions, it has a good C statistic value, has been externally validated (51) and contains only pre-procedural information.

Despite the increasing complexity of stratification models, prior chronic kidney disease remains a substantial independent risk factor for CI-AKI development and is the most prevalent risk factor in most stratification models (53). Recently, Serif et al. published a prospective assessment of the diagnostic performance of available risk stratification systems until the year 2018 for CI-AKI prediction (51). The predictive ability of stratification models proved to be only modest. None of the models passed past C-statistic ≥0.8. Yin et al. (54) later assessed the calibration and discriminatory value of risk scores on patients undergoing percutaneous coronary intervention or coronary angiography. The best predictive value according to Yin et al. has the risk model by Chen et al. (55). In most studies with risk prediction models, CI-AKI “narrow” definition is used (increase in Serum Creatinine of 44.2 μmol/L). For reference see Tables 1, 2.

We conceptualized two tables (Tables 3, 4) depicting those risk factors with the sufficient strength of evidence for better visualization of current proven and unproven risk factors of CI-AKI.

**TABLE 3:** Proven risk factors of CI-AKI.

Patient-related risk factors—modifiable	Weight of evidence	Evidence
Anemia	Systematic review and meta-analysis	Wei et al. (191)
Hypotension	Systematic review and meta-analysis	Ong et al. (192)
Volume Depletion and Hemodynamic Instability	Systematic review and meta-analysis	Tea et al. (193)
NSAID, diuretics and other nephrotoxic drugs	Systematic review and meta-analysis	Zhang et al. (194)
ACE-I/ARB usage	Randomized trial parallel-group, single-center	CAPTAIN trial (195)
Acute heart failure NYHA III-IV	Multicenter retrospective study	Tian et al. (196)
Hypoalbuminemia	Systematic review and meta-analysis	Liwei et al. (197)
Peri-procedural hyperglycemia	Systematic review and meta-analysis	Jakrin et al. (198)

**Patient-related risk factors—unmodifiable**	**Weight of evidence**	**Evidence**

Chronic kidney disease < 60 ml/min/1,73 m^2^	Retrospective analysis of multicenter national registry on ACS	Neves et al. (199)
Diabetes mellitus	Systematic review and meta-analysis	Liu et al. (200)
Age > 70–75 years	Meta-analysis	Wei et al. (18)
Acute coronary syndrome KILIP III-IV	Retrospective single center study	Jain et al. (201)

**Procedure related risk factors**	**Weight of evidence**	**Evidence**

Femoral approach	Systematic review and meta-analysis	Andó et al. (202)
Repeated exposure of contrast agent within 72 h	Retrospective analysis of multicenter data from Texas Quality Initiative	Tecson et al. (203)
Volume of CM over MACD	Multicentric, regional retrospective analysis	Gurm et al. (204)
Emergent/urgent procedure	Multiple single and multicentric randomized trials	Isaac et al. (193)
IABP use	Retrospective analysis of patient dataset	Mehran et al. (49)

CM, contrast media; MACD, maximum allowable contrast dose; IABP, intraaortic balloon pump.

**TABLE 4:** Unproven risk factors of CI-AKI.

Procedure related risk factors	Weight of evidence	Results
Iso-osmolar instead of low osmolar CM.	Systematic review and meta-analysis	Solomon et al. (205)
NaHCO 3 instead of NaCl solution	Systematic and meta-analysis (not superior to NaCl)	Zapata-Chica et al. (206)
Timing of Angiography with cardiac surgery (detrimental if less than 7 days apart)	Systematic review and meta-analysis	Liu et al. (207)

**Patient-related risk factors—modifiable**	**Weight of evidence**	**Evidence**

Hypertension-Inconclusive findings	Metanalysis	Inconclusive
Patient-related risk factors—unmodifiable	Weight of evidence	Evidence
Monoclonal gammopathies	Systematic review and meta-analysis	Stacul et al. (208)
Female sex -Inconclusive findings	Systematic review and meta-analysis	Neugarten et al.

CM, contrast media.

### New markers of contrast-induced acute kidney injury

As a result of the inherent kinetics of creatinine, CI-AKI is not diagnosed sufficiently early due to its slow rise. Creatinine will gradually increase within 24–48 h after renal injury and is modulated by several factors such as age, sex, muscle mass, and medication. Cystatin C levels increase more rapidly, compared to creatinine, although it is affected by non-GFR factors as well. Therefore, it would be helpful to identify new biomarkers that could be used to diagnose CI-AKI earlier.

#### Cell cycle arrest biomarkers

As a result of progress in the theory and practice of proteomics, several proteins have been investigated as potential biomarkers of CI-AKI. Namely, Neutrophil gelatinase-associated lipocalin (NGAL), insulin-like growth factor-binding protein-7 (IGFBP-7), tissue inhibitor of metalloproteinase-2 (TIMP-2), Interleukin 18 (IL-18), liver fatty acid-binding protein (L-FABP), and Kidney injury molecule (KIM-1) have been investigated. Among those, the combination of TIMP-2 and IGFBP 7 proved to be superior to others (56, 57). Both IGFBP7 and TIMP-2 are inducers of G1 cell cycle arrest. When renal cells encounter stress or insult, it expresses those proteins in an early phase of injury. Expression of those proteins leads to the arrest of the proliferation of tubular renal cells. Therefore, increases in TIMP 2 and IGFBP7 blood levels serve as an indication of cell-cycle arrest in renal parenchyma cells as a means of protection when exposed to stress (such as iodinated-CM). When these biochemical markers are used in combination with [TIMP-2] × [IGFBP7] cut-off value of >0.3 (ng/ml)^2^/1,000 the sensitivity and negative predictive value are extremely high. Opal Study showed a sensitivity value of 89% and a negative predictive value of 97% (58). Cell cycle arrest biomarkers could also be applied to non-cardiac surgeries (59) or in settings of sepsis or cardiac arrest (60).

Due to the positive results from numerous studies, FAD approved the use of [TIMP-2 x IGFBP 7] for early prediction of AKI in clinical settings. This new test is called Nephrocheck ^®^ (Astute Medical, San Diego, CA, USA) and was one of the first commercially available tests of cell cycle arrest biomarkers in urine. The benefits of such a system are apparent in the quick turnaround time of 20 min, availability as a point-of-care test and requirement of small amounts of urine for diagnostic purposes. In current clinical practice, values of 0.3 (ng/ml)^2^/1,000 require a nephrology consult due to the high risk of CI-AKI (56).

Animal trials published to date suggest that not only the induced cell-cycle arrest of kidney cells is peri-procedurally possible, but it also seems beneficial. The manipulation of cell-cycle may represent a new therapeutic strategy in the prevention and treatment of AKI. (61). Thus, cell-cycle arrest activation and deactivation at critical clinical time may prove to be a therapeutic intervention in the future.

#### Novel protein biomarkers

Another known novel protein used as a biomarker is NGAL. NGAL is upregulated in parts of the nephron as a reaction to AKI. This leads to an increase in urinary and plasma volumes of NGAL. Diagnostic systems capable of performing NGAL assays are ARCHITECT^®^ and ALINITY^®^ I Urine NGAL assays (Abbott) and BioPorto^®^ NGAL test (BioPorto Diagnostics). Inconveniently cut-off value indicating the presence of the renal disorder is not known for ARCHITECT^®^ and ALINITY^®^, however higher values are predictive of renal injury and subsequent AKI development. The meta-analysis by Yi He (62) et al. and Lareyere et al. (63) showed non-inferior results compared to cystatin C performance. Compared with IL-18, KIM-1, L-FABP, CRP, and BNP, NGAL exhibited increased predictive value in CI-AKI after PCI (64). However, Sharrod-Cole et al. reported the utility of plasma NGAL in patients undergoing CABG as inconclusive of CI-AKI prediction (65).

Recent meta-analysis about the performance of Kidney injury molecule 1(KIM-1) was published. Following renal injury or stress, KIM-1 gets upregulated and facilitates recognition and clean-up of dead renal cells in the injured kidney. Therefore urinary KIM-1 values has high predictive value for the diagnosis of CI-AKI in patients undergoing administration of iodinated CM (66).

Concerning those discoveries, ongoing trials focused on thorough examination of those novel biomarkers may result in the subsequent implementation of the routine level assessment of these into clinical practice.

#### Micro RNAs

Serum creatinine and Serum Cystatin C, the most known markers for CI-AKI are elevated only at the later stage of CI-AKI. The search for biomarkers with earlier peak values is therefore of high importance. Current evidence suggests the applicability of MicroRNA (miR) measurement in predicting CI-AKI development. MiR work as regulatory agents that binds to mRNAs and degrade them. It was demonstrated that changes in levels of miRNA correlate to acute and chronic kidney injuries (67). The most recent comprehensive meta-analysis demonstrated that upregulation in specific miRs (namely miR 30a, miR-30ce) could be used as a novel biomarker for CI-AKI (68). MiR values rise in 4–6 h post-CM administration while the serum concentration of creatinine may take 24–36 h to rise after a renal insult. Specifically, miR could also be targeted for potential direct therapy, namely miR-188 activation attenuation or a conception of its antagonist could be used for future. These particle levels are exacerbated in apoptosis induced by CI-AKI (69). Already mentioned overexpression of miRNA-188 that induces apoptosis by upregulating SRSF7 gene may be a target therapy for CI-AKI. As of today, the main limitations for the routine use of these are high costs and limited availability of capable laboratories. More randomized clinical trials are needed for the definitive evidence.

We advocate adopting models presented by Chen et al. or Gurm et al. due to the external evaluation and exclusively incorporated pre-procedural risk factors. However, considering currently published breakthroughs, we propose that a new model should be developed. Hematologic and biochemical factors such as neutrophil/lymphocyte ratio, platelet/lymphocyte ratio, red blood cell distribution width, and hsCRP could also be included in this model. These values are normally known ahead of time or are simply calculated, and they do not incur any additional expenses.

A more prudent method would be to test novel biomarkers such NGAL, IGFBP-7, and TIMP-2 to predict and detect CI-AKI earlier (70).

In conclusion, a new risk prediction model for practical CI-AKI risk stratification is needed. External validation of models, as well as generalizability to all diagnostic procedures with CM administration, is an important next step in the development of stratification models. However, the true applicability of current models is questionable in emergencies such as STEMI or Cardiogenic shock, where laboratory results or prior medical history is usually not known. Therefore, it is needed to have an easily applicable risk model for those situations as well. We would also recommend an internet-based, app-based, or hospital software-implemented calculator because more sophisticated models are frequently more sensitive while being more difficult to calculate on the spot.

## Strategies to prevent contrast-induced acute kidney injury

### Selection of contrast media and dose reduction

According to the published data on CI-AKI prevention, minimizing the use of iodinated contrast agents and using low or iso-osmolar CM agents appears to be of greatest importance (71, 72). These recommendations are included in the recent guidelines by both the European Society of Cardiology (73) and the American College of Cardiology (74).

In modern interventional and diagnostic procedures, the most used CM is low-osmolar non-ionic CM or iso-osmolar CM (specifically iodixanol). Low osmolar CM was found to be non-inferior to the iso-osmolar CM (75). Since the choice of CM is relatively limited and since the CI-AKI risk depends on CM volume, the interest of interventional cardiologists is presently focused on CM volume reduction. At the same time, to maintain the effectivity of invasive and interventional procedures, innovative imaging techniques may be required.

### Intravenous hydration

CM administration leads to an increase in urine viscosity proportional to CM volume. Animal and human studies showed that sufficient hydration reduces urine viscosity after CM administration (27, 76). Therefore, periprocedural hydration is one of the preventive measures listed in the ESC and ACC practice guidelines (73, 74). The proof of efficient CI-AKI prevention by intravenous isotonic fluid hydration has also been reported in numerous studies and meta-analyses (see Table 5). However, Jiang et al. (77) reported that the hydration effect appears to be lower during less severe CKD stages. The effectivity of sodium bicarbonate had long been discussed before the PRESERVE study, which found no benefit or harm compared to isotonic saline (78). The advantages of other balanced fluids, such as Ringer’s lactate instead of saline solution, remain presently unproven. Studies in critically ill patients reported preference of Ringer’s lactate to other solutions due to the danger of hyperchloremic acidosis (79, 80). Nevertheless, the BASICS trial did not confirm this because no 90-day mortality difference was found between balanced solution and 0.9% saline solution (81).

**TABLE 5:** Randomized controlled trials comparing hydration as prevention method of CI AKI.

Name of study	Type of study	Type of population	Inclusion criteria (renal functions)	Number of patients	Type of intervention	CI-AKI incidence (control/study)
AMACING (189)	RCT-monocentric	Elective procedure with i.v. CM (only 16% intraarterial)	60 ml/min per 1⋅73 m^2^ or lower	603	1–4 ml/kg/h of saline to no iv. fluid admitted	2.6% (8/307) 2.7% (8/296)
POSEIDON (82)	RCT-monocentric	Indication of PCI	60 ml/min per 1⋅73 m^2^ or lower	350	LVEDP-guided volume expansion vs. standard fluid administration protocol	16.3% (28/172)/ 6.7% (12/178)
Jurado-Román et al. (190)	RCT-monocentric	STEMI patients	Not mentioned-unrestricted	408	Saline fluid 1ml/kg/h for 24h vs. no iv. fluid admitted	10.8% (22 of 204)/ 21.1% (43/204)/

RCT, randomized clinical trial; LVEDP, left ventricular end diastolic pressure, iv., intravenous; PCI, percutaneous coronary intervention.

Uncertainty remains in respect of the appropriate volume of fluids to be administered. This question was partially answered by the randomized POSEIDON trial (82). CI-AKI occurred less often in patients with left ventricular end-diastolic pressure (LVEDP) guided hydration group (6.7%) compared to standard hydration protocol in the control group (16.3%). POSEIDON trial also supplied the first evidence that patients tolerate and benefit from periprocedural fluid administration above 5 ml/kg/h. The POSEIDON trial findings were confirmed in the meta-analysis by Michel et al. (83) which showed that patients benefit from the intensive in comparison to the conservative hydration regime.

These findings led to the development of devices specifically designed to force diuresis. The principles of such tools also correspond to the older findings of the PRINCE study which showed that maintaining steady high urine flow effectively eliminates CM (84). The first device utilizing such a technique was the RenalGuard™(CardioRenal Systems, Milford, MA, USA). RenalGuard™ uses standard urinary Foley catheter and closed loop fluid replacement system to adequately replace fluid in real time. The balance between the fluid intake and the forced diuresis output proved to be superior to the saline hydration alone as shown in the REMEDIAL II (85) and MYTHOS (86) trials, and subsequently also proved to be superior to the LVEDP guided hydration, as shown in the REMEDIAL III trial (87). Drawback of the device includes the initial costs of the system.

The comparison of meta-analyses of iv. hydration regimes have been visualized in Table 6.

**TABLE 6:** Comparing meta-analysis of intravascular volume expansion as a prevention method for contrast associated acute kidney injury.

Author of metanalysis	Number of RCTs included	CI-AKI incidence (RR)	Dialysis or mortality incidence
Michel et al. (83)	37	intensive volume expansion strategies associated with a 34% risk reduction	Remains unchanged
Wang et al. (209)	3	Intensive volume expansion strategies associated with a 36% risk reduction	Remains unchanged
Liu et al. (210)	3	Intensive volume expansion strategies associated with a 42% risk reduction	Remains unchanged
Jiang et al. (77)	7	Risk reduction of CI AKI mostly in severe CKD defined as 30 ml/min 1.72 m^2^	Remains unchanged

RR, risk reduction; RCT, randomized clinical trial; CKD, chronic kidney disease.

#### Bioimpedance vector analysis guided hydration

As previously stated, intravenous volume expansion is the mainstay of preventative methods. There is, however, no simple and effective non-invasive way for assessing current fluid status and its minute changes. Bioimpedance vector analysis (BIVA) is an accurate tool for determining the patient’s current extracellular water volume which closely correspond to intravascular volume. Maioli et al. reported a study that used BIVA analysis on patients undergoing PCI for stable coronary artery disease. Lower BIVA fluid status was an independent and significant predictor of CI-AKI. (88). This insight was applied in the BELIEVE pilot study, which used BIVA to guide volume expansions. Despite the smaller number of patients (*n* = 61), the BIVA-guided hydration group had a decreased incidence of CI-AKI (3.3% vs 6.5%). It did not, however, achieve statistical significance (89). Maioli et al. conducted the largest RCT to date. Patients were classified as having a low or normal bodily fluid level based on their BIVA levels. Patients with low body fluid were randomly assigned to periprocedural hydration of 1–2 ml/kg/h. Patients receiving 2 ml/kg/h saline had a lower incidence of CI-AKI than those getting standard amount of saline (11.5% vs 22.3%, *p* = 0.015) (90). Therefore, measuring BIVA levels could aid in the regulation of intravascular volume expansion and potentially minimize the occurrence of CI-AKI.

### N-acetylcysteine

Acetylcysteine (NAC) intravenous or oral administration was among the first methods proposed for the CI-AKI prevention (91). One of the CM consequences is the accumulation of reactive oxygen species (ROS) and their adverse effect on the renal parenchyma (44). ROS directly affects cellular proteins and organelles membranes by generating highly reactive molecules (e.g., hydrogen peroxide, hydroxyl radicals, and superoxides) (44). Those molecules act indirectly in the regulation of microcirculation by creating vasoconstrictors (e.g., endothelin and angiotensin II) (92). NAC acts as a potent antioxidant that disrupts these pathogenetic mechanisms. NAC also prevents urinary nitric oxide uptake after angiography (93).

Studies that compared NAC as addon therapy with concomitant Iv., preferably saline, hydration are listed in Table 7. Search criteria from section “Methods” was used. We choose to omit trials that had less than 200 patients due to low statistical power. We also omitted intrarenal or other non-standard application of NAC.

**TABLE 7:** RCT comparing N acetyl cysteine as prevention method of CI-AKI.

Name of study	Type of study	Type of population	Inclusion criteria (renal functions)	Number of patients	Type of intervention	CI-AKI incidence (study/control)
PRESERVE Trial (78)	RCT, multicentric	Indication for coronary or non-coronary angiography	eGFR 15–44 ml/min/1.73m^2^ or 45-59.9 ml/min/m2 with DM	4,993	FLUID (Saline or NaHCO_3_) + NAC, Total dose of 12,000 mg, oral.	4.6% (114/2495)/4.5%(112/2498)
ACT Trial (211)	RCT, multicentric	Coronary or peripheral arterial diagnostic angiography or PCI	eGFR ≤ 60 ml/min/m^2^	1,369	FLUID + NAC, total dose 4,800 mg, intravenous	13.8% (97/702)/14.7% (98/667)
LIPSIA-N-ACC Trial (212)	RCT, monocentric	STEMI patients undergoing primary PCI	Not mentioned-unrestricted	251	FLUID + NAC, total dose 6,000 mg, intravenous	14% (18/126)/20% (25/125)
CASIS Trial (213)	RCT, multicentric	Indication for coronary angiography or/and PCI	eGFR ≤ 60 ml/min/m2	220	Fluid or high dose fluid + NAC total dose 2.400 mg), intravenous	2.5% (2/80)/16.3% (13/80)/ 10% (6/60)
Thayssen et al. (214)	RCT, multicentric	STEMI patients undergoing primary PCI	Not mentioned-unrestricted	720	Fluid with NAC or NAHCO3 or COMBINED	20.1% (32/176)/20.1% (33/181)/20.1% 33/177 (20.8%)/26.5% (43/181)
Carbonell et al. (215)	RCT, monocentric	Indication for coronary angiography or/and PCI	eGFR > 60 ml/min/m^2^	216	Fluid + NAC, Total dose of 2.400 mg, intravenous	10.2% (11/107)/10,1% (11/109)
Kay et al. (216)	RCT, Monocentric	Indication for coronary angiography or/and PCI	eGFR > 60 ml/min/m^2^	200	FLUID + NAC, Total dose of 2,400 mg	4% (4/102)/12% (12/98)
Jaffery et al. (217)	RCT, Monocentric	ACS undergoing coronary angiography and/or PCI	Not mentioned-unrestricted	398	FLUID + NAC, total dose of 6,000 mg, intravenous	16% (33/206)/13% (25/19)
Marenzi et al. (218)	RCT, Monocentric	STEMI patients undergoing primary PCI	Not mentioned-unrestricted	354	Fluid + NAC or HIGH DOSE NAC, Total dose of 3,000–6,000 mg, intravenous	8% (10/118)/15% (17/115)/33% (39/119)
Reinecke et al. (132)	RCT, Monocentric	Elective left heart catheterization	sCr ≥ 1.3 mg/dl and ≤ 3.5 mg/dl	412	Fluid + NAC, total dose of 2,400 mg	2.9% (4/140)/5.8% (8/137) (Omitting hemodialysis int. arm)

eGFR, estimated glomerular filtration rate; NAC, N acetylcysteine; RCT, randomized clinical trial; Control, iv hydration alone with saline; sCr, serum creatinine.

The NAC doses ranged from 2.500 to 15.000 mg over 2–4 days. Adverse effects of NAC are well known from paracetamol detoxification (94) and commonly include flushing, pruritus and rash. Their onset is rapid, and incidence range between 10 and 15%. Severe reactions, such as bronchospasm or hypotension with a need for epinephrine application, are relatively rare ranging around 1–5% (95, 96).

Studies and trials of CI-AKI prevention by NAC resulted in discordant and uncertain results. The diversity of these results might be explained by different patient populations, variable dosing regimens, and/or concomitant infusions of balanced solutions. Follow-up periods were also not consistent. Despite the absence of a clear benefit of periprocedural NAC administration, the practice appears continued across medical centers. This might be contributed by NAC safety, low cost, and application ease. Nevertheless, considering the discordant observations, NAC administration is not recommended as the sole intervention for CI-AKI prevention (6).

Nevertheless, this recommendation is not entirely consistent with the recent meta-analyses (Table 4). Largest meta-analysis of the 61 randomized clinical trials was performed by Xu et al. (97). The incidence of CI-AKI was 12.8% in the NAC group vs. 16.0% in the control group leading to a highly significant difference. Specifically, in patients undergoing coronary angiography, the incidence of CIN in the NAC and control groups was 13.7 and 17.2%, respectively; again, highly statistically significant. In patients undergoing computed tomography, the corresponding incidences were 7.7, 14.8%, respectively, while no difference was observed in patients undergoing peripheral angiography. The NAC effectiveness also demonstrated in the meta-analysis of 49 randomized clinical trials by Sadegh et al. (98).

Given the overall positive findings in the meta-analyses (see Table 8), NAC need be thoroughly examined in properly designed multicenter studies, mainly investigating NAC as an add-on therapy rather than a standalone CI-AKI prevention method.

**TABLE 8:** Comparing meta-analysis of NAC treatment as a prevention method for contrast associated acute kidney injury.

Author of metanalysis	Number of RCTs included	CI-AKI incidence (RR)	Dialysis or mortality incidence
Ali-Hasan-Al-Saegh et al. (98)	49	21 % lower risk of CIN than the control group	Remains unchanged
Xu et al. (97)	47	24 % lower risk of CIN than the control group	Remains unchanged

RR, relative risk reduction; RCT, randomised clinical trial; NAC, N-acetylcysteine; RR, risk reduction in%.

### Statin therapy

Retrospective and prospective studies have shown that statins have a protective effect in preventing contrast-associated nephropathy (99). These findings were confirmed by recent meta-analyses (100, 101). Effects of statins range from anti-inflammatory effect to vasodilator modulation, and inhibition of pro-apoptotic processes (100, 102, 103). These processes are likely responsible for the alleviation of endothelial dysfunction, increase of nitric oxide availability to the renal capillary endothelium, and inflammatory response inhibition (104). The statin-induced inhibition of the inflammatory response, measured by a decrease in hs-C-reactive protein, was demonstrated in the JUPITER study (105). Statins also reduce the number of endothelial angiotensin receptors and inhibit endothelin synthesis. These changes might also reduce the CI-AKI incidence (106).

Second or third-generation statins have been used in most randomized clinical trials investigating their effect in CI-AKI prevention. Specifically, rosuvastatin, atorvastatin, or simvastatin were used at doses corresponding to high-dose intensity. This corresponds to 20–40 mg for rosuvastatin and 40–80 mg for atorvastatin or simvastatin. The duration of treatment ranged from hours to days after the intervention or inclusion of patient into the trial.

One of the first clinical trials investigating statin therapy for CI-AKI prevention was the PROMISS study (107). However, this study has not found any CI-AKI prevention benefit of simvastatin in comparison to placebo (2.5% vs 3.4%). Conversely, the PRATO-ACS (71) study found a statistically significant reduction of CI-AKI incidence on rosuvastatin compared to placebo (6.7% vs. 15.1%). TRACK D study (108) reported similar findings when using a larger cohort. PRATO ACS-2 randomized trial further demonstrated that atorvastatin was non-inferior to rosuvastatin in preventing CI-AKI (109).

Some systematic meta-analyses have recently reviewed the use of statins as a CI-AKI prevention method (see Table 9). Their results were generally positive; however, one of them showed no effect on incidence of CI-AKI. These heterogeneity of individual RCT protocols prevents statins to be universally recommended for CI-AKI prevention.

**TABLE 9:** Comparing meta-analysis of statin therapy as prevention method for CI-AKI.

Author of metanalysis	Number of RCTs included	CI-AKI incidence (RR)	Dialysis or mortality incidence
Zhang et al. (219)	12	Slight risk reduction of no statistical significance.	Remains unchanged
Liu et al. (220)	9	Risk reduction of CI AKI was 53%	Remains unchanged
Khan et al. (101)	14	Risk reduction of CI AKI was 47%	Remains unchanged
Cho et al. (100)	8	Risk reduction of CI AKI was 59%	Remains unchanged

RR, risk reduction; RCT, randomized clinical trial.

A multicenter randomized clinical trial is therefore needed to address the protective CI-AKI effect of high-intensity statins in patients undergoing interventional cardiology and other imaging procedures. Notwithstanding this need, the recent clinical practice guidelines uniformly indicate high-intensity statins for atherosclerotic disease. Therefore, almost all cardiac patients undergoing imaging procedures will be on HMG-CoA reductase inhibiting drugs.

### Other pharmacologic prevention

Many other agents that have been evaluated to prevent CI-AKI. Anti-ischemic agent trimetazidine (110) has been reported to possess reno-protective effects due to oxidative stress attenuation, inflammatory cytokine release, and oxygen and energy balance maintenance (80). Other agents evaluated for CI-AKI prevention include nifedipine (111), alprostadil (41), dopamine agonist fenoldopam (112), atrial natriuretic peptide (113), and vitamin C (114). Importantly, most of the studies investigated these agents in combination with intravenous hydration. Despite positive result from monocentric study (115), the results were not replicated nor supported in adequately powered trials and meta-analyses (116). None of these additional pharmacologic preventions are currently widely accepted for CI-AKI prevention.

Recently, trials with an SGLT2 inhibitor as a possible treatment for CI-AKI have been published. Patients who had had PCI with an SGLT2 inhibitor had a smaller increase in creatinine levels and, as a result, a reduced incidence of CI-AKI (3.8% vs 17.3%, *p* = 0.05) (117) (118). In a recent animal investigation, SGLT2 inhibitor was shown to have positive effects on rat renal cells, including a 57.6% decrease in the frequency of apoptotic cells and the suppression of oxidative stress inducers (HIF-/HE4/NF-B pathway) (119).

Current guidelines support interruption of metformin before the CM procedure, especially in patients with kidney impairment (eGFR ≤ 30 ml/min/1.73m^2^) (6, 73). Interruption of other nephrotoxic medication appears to have a limited value, while ACE-I/ARB cessation was found to be beneficial in recent trials and metanalyses (117, 120). Given the current positive results from SGLT2 inhibitor trials, larger prospective randomized trials with SGLT2 inhibitors may result in a standard pharmacologic CI-AKI prevention in the future.

#### Heme oxygenase 1

Heme-oxygenase is an inducible enzyme with anti-apoptotic and antioxidant properties. Its antioxidant properties are explained by the overexpression of bile pigments, which scavenge free radicals, and by trapping hydrogen peroxide in the biliverdin-to-bilirubin conversion reaction. Carbon monoxide is another by-product with anti-inflammatory properties (121). Animal studies demonstrated the effectiveness of HO-1 inducer (hemin) as an innovative strategy for CI-AKI mitigation, as evidenced by a decrease in CI-AKI novel biomarkers and alleviation of oxidative stress (122). Heme-oxygenase could be induced by a variety of agents, including heme arginate, hemin, and a nrf2 activator, with overall positive results in HO-1 increase. HO-1 inductions have also been tested in other areas of expertise; specifically, a protective effect was demonstrated in kidney transplantation settings with a reduction in the incidence of acute rejection or sepsis (121, 123). Although the results appear promising, more research is needed to determine whether HO-1 upregulation protects against AKI when administered after the injury. Given the inherent toxicity of bile pigments and CO (if significantly increased), the optimal dose has yet to be determined. The development of non-toxic HO-1 inducers is also critical. Regardless of the challenges, Heme-oxygenase induction is the promising field of study and potential prevention method that should be investigated further.

### Remote ischemic preconditioning

Remote ischemic precondition (RIPC) is a phenomenon in which episodes of transient ischemia on distal organs provide the resistance to the subsequent episode of ischemia on central organ. It was demonstrated that the brief periods of ischemia to muscular muscle protect kidney and cardiac tissue (124). Interestingly, benefits of RIPC from one animal could be transferred to another through transfusion of plasma. Current evidence from literature indicates that responsible particles could be the micro-RNAs (125). Therefore, identification of those particles could enable simple prevention pharmacological therapy for CI-AKI.

Number of studies examined whether RIPC may be able to protect the kidney. Wever et al. investigated whether cycles of brief limb occlusion by manual sphygmomanometer can protect against renal ischemia-reperfusion injury. Bilateral RIPC was more effective than unilateral RIPC, but both improved renal function by 30–60% (126). Effect of RIPC was further proved in Renal Protection Trial (127). Meta-analysis by Deng et al. showed that RIPC can reduce risk of CI-AKI in patients undergoing PCI/Coronarography (128). Recent up to date meta-analysis showed that RIPC reduced incidence of AKI in patients undergoing cardiac surgery. This effect was only visible in patients undergoing general anesthesia through volatile gases (129).

Although many new studies have further advanced our knowledge in this area, the appropriate intensity of RIPC and its mechanisms of action are still unknown. RIPC is a safe procedure, as no relevant adverse events related to RIPC application were reported in the clinical trials performed to date. This approach may offer a novel, non-invasive and inexpensive treatment strategy for decreasing AKI incidence in high-risk patients.

### Renal replacement therapy

Hemodialysis can remove CM from the circulation and its role in the treatment of kidney failure is well established. However, this does not apply to CI-AKI prevention (130). Despite some positive results from the older studies (131), preventive hemodialysis is not generally accepted for this purpose (105, 132). Inappropriate timing or inadequate ultrafiltration might have been the reasons for the failure of preventing CI-AKI by hemodialysis. Even in chronic hemodialysis or peritoneal dialysis patients, the additional procedure after contrast injection is not recommended (6, 133). Choi et al. (134) investigated simultaneous hemofiltration (performed only during angiography) during coronary interventions. Despite logistic challenges, his study showed better late-stage renal protection against CI-AKI than periprocedural hemofiltration (6 h pre- and 24 h post-angiography).

### Contrast modulation and alternative imaging modalities

Innovative and/or alternative imaging techniques might assist in CI-AKI avoidance attempts. Specific techniques have been proposed to reduce the CM volume. These include including CM dilution, contrast modulation systems, intravascular ultrasound (IVUS), and non-contrast optical coherent tomography (OCT) based-guided PCI.

#### Contrast dilution

The most basic method for contrast volume reduction is CM saline dilution. It has been proposed that dilution of up to 50% could be used without compromising imaging quality (135). In few studies, the limit of dilution is even higher, Takumi Kuriyama et al. achieved contrast dilution of 20% (136). However, this study was done in patients undergoing determination of cerebral blood volume by flat-panel detector imaging (FD-PBV). Another method for contrast reduction is automated contrast injectors (ACI) that are being slowly adapted into clinical routine. This device enables to deliver smaller amount of CM compared to manual injections. Results from meta-analysis by Minsinger et al. proved that ACIs could reduce CM volume by 45 ml and reduce CI-AKI incidence by 15% (137). ACI can be used with DyeVert™ system (specifically, DyeVert Power XT system) to enhance the CM volume reduction further (138).

#### Contrast modulation or removal

The volume of CM can be minimized, provided an ideal coronary artery visualization. This can be achieved by limiting coronary reflux through optimized delivery profile of CM and minimized excess aortic reflux. The emerging methods for CM volume reduction are lately being investigated.

Most notably, FDA-cleared a dedicated contrast-reducing DyeVert™ system (Osprey Medical, Minnetonka, MN, USA). It’s a device intended to reduce CM volume while maintaining image quality. It available in options compatible with manual as well as automated contrast injections.

AVERT trial was the first randomized study that investigated a dedicated contrast reducing DyeVert™. Although the AVERT system was feasible, safe and significantly reduced CM volume (15.5% relative reduction overall, and 22.6% among PCI patients), no significant differences in CI-AKI incidence were observed compared to periprocedural hydration alone (139). On the contrary, Briguori et al. recently reported positive effect by the same DyeVert™ system in a non-randomized study of acute coronary syndrome patients. In this study, the device reduced the CM volume by 38% and was associated with a statistically significant CI-AKI decrease compared to conventional manual injection syringe with periprocedural hydration (8% vs. 19%) (140). Tarantini et al. recently conducted a meta-analysis on 17 studies using the DyeVert™ system in patients undergoing coronary angiography or PCI. The contrast volume was reduced by 39.7% in this study. The DyeVert™ system cohort had an absolute risk reduction of 5% (CI, 0.40–9.80%; *p* = 0.03). As a result, the number needed to treat for CI-AKI prevention is 20, preserving adequate image quality in 98% patients (141). Further information from a UK-based Cost-utility Analysis revealed that DyeVert™ results in cost savings as well as improved effectiveness over the course of a patient’s lifetime (−£435 and +0.028 QALYs) (142). The ongoing Renal Insufficiency Following Contrast Media Administration Trial IV (REMEDIAL IV) seeks to determine whether the DyeVert™ system is effective in lowering the rate of CI-AKI in patients with acute coronary syndrome undergoing urgent/immediate (within 2 h) invasive diagnostic or interventional cardiovascular procedures. The ongoing DyeMINISH Registry aims to compare CI-AKI incidence and MACE both with and without DyeVert™. Patient enrollment is expected to be completed in late 2023.

Another alternative is to remove the CM directly from the coronary sinus. Because CM is removed from the myocardium via the coronary sinus, direct engagement, and the aspiration of blood from the coronary sinus may reduce peri-procedural CI-AKI. One of the initial studies used CINCOR™, an 11-French modified catheter. Although this system can reduce administered CM volume by 27% without affecting hemoglobin levels, it requires a femoral approach (a proven risk factor for CI-AKI) (143). Similarly, Diab et al. removed approximately 40% of the given CM. Consequently, the incidence of CI-AKI was reduced by a factor of five in this study (144). Another approach is to engage the coronary sinus via a two-lumen balloon-tipped catheter with multiple side holes. Following successful balloon inflation to block the inflow of CM to the coronary sinus, venous blood was aspirated. This feasibility study resulted in a decrease in overall CM volume with no changes in hemodynamic variables (145).

#### Intravascular ultrasound-guided percutaneous coronary intervention

With the help of IVUS, ultra-low or even “zero-contrast” PCI is possible to perform by adequately trained and experienced operators (146, 147). Unlike standard OCT, there is no need for iodinated CM. Therefore, IVUS appears particularly appropriate for guiding PCI in patients with chronic kidney disease or at high CI-AKI risk although it has been successfully used also in other patients (78, 106). Extremely low volume of contrast media can be achieved to be administered using this technique. The technique may safely be used in calcified, tortuous, or grafted veins and arteries (146). Peri-procedural IVUS is feasible and reduces CM volume and radiation exposure in patients undergoing chronic total occlusion procedures. (148). Data from a single-center RCT of CKD patients undergoing IVUS guided PCI were recently published. Although this study demonstrated the feasibility and safety of zero-contrast PCI, it lacked a comparative group and CI-AKI incidence was 11.9% (149). The authors speculate that it could be attributable to the femoral approach and subsequent atheromatous embolization of renal arteries, or to the *ad hoc* nature of the procedure. Therefore CI-AKI incidence reduction by IVUS-guided PCI compared to conventional angiography-guided PCI still needs to be proved in properly designed randomized trials, e.g., in the ongoing MOZART II trial (150).

#### Optical coherent tomography-guided percutaneous coronary intervention with different media

Optical coherence tomography uses a near-infrared light for image acquisition of the coronary vessel wall with the higher axial resolution compared to angiography and/or IVUS. However, high-quality OCT requires iodinated CM flush and complete blood clearance during image acquisition. The use in patients prone to CI-AKI is consequently limited. There is thus need for an alternative medium but neither low-molecular-weight dextran (LMWD) (151), nor heparinised saline (152), nor a mixture of saline and colloid (153) are ideal. Notably, LMWD is limited by its cost and slight risk of anaphylactoid reaction (154). Anuric renal failure has also been reported following LMWD administration, though this has recently been dismissed (151). Heparinised saline resulted in more speckling and smaller area and diameter values compared to iodinated CM OCT as pointed out in animals study (155). The comparison of IVUS and OCT guided PCI and both pros and cons of those techniques are in Table 10.

**TABLE 10:** Comparison of IVUS and OCT applicability and attributes.

Attributes	IVUS	OCT
Requires blood clearance by CM	**−**	**+**
Spatial resolution	**−**	**+**
Full vessel thickness visibility	**+**	**−**
Aorto-ostial lesion visualization	**+**	**−**
Reproducibility of image	**+**	**+-**

#### Carbon dioxide guided angiography

An alternative to conventional angiography using iodinated-CM is carbon dioxide angiography. The availability of this method in patients with CKD or allergy to iodinated CM has been facilitated by the creation of precise CO_2_ delivery systems. No documented adverse renal effects make it the perfect substitute in patients with CKD undergoing interventional diagnostic procedures. The fundamental restriction in current practice is that CO_2_ should not be utilized in arteries above the diaphragm (such as renal and mesenteric arteriography, venography below the waist) due to the potential risk of cerebral air embolism. Although this complication is uncommon (affecting less than 1% of all CO_2_ patients), it can theoretically lead to stroke and death (156). Recent animal studies, however, have demonstrated that CO_2_ coronary angiography is feasible and has no negative effects on animal health, particularly when used in conjunction with an automated injector that avoids gas reflux, such as Angiodroid (Angiodroid Srl, Bologna, Italy) (157). Another complication may be a vasospasm caused by the rapid injection of CO_2_ gas, which can be alleviated by prolonged administration of CO_2_ (158). CO_2_ could currently be used to detect endovascular leaks during EVAR as an alternative to iodine-containing CM (159). Recently, promising results from studies in patients undergoing endovascular procedures for peripheral artery disease have been published. According to these studies, patients with CKD 4-5 should be treated primarily with CO2-guided endovascular procedures revascularization (160).

#### Alternative techniques for visualization of coronary tree

A novel technology for real-time live navigational guidance in coronary tree was recently patented by Philips. It is called Dynamic coronary roadmap™ (DCR) and was designed for procedure efficiency in complex PCI. This is achieved due to the overlaying automatically generated map of coronary tree vessels which are superimposed on live fluoroscopic images. First clinical use was reported in 2015 (161). Hereafter feasibility study was performed on 36 patients undergoing PCI with support of DCR™. In 99.5% the quality of roadmap was fit for general use (162). This led to the first prospective open-label trial with DCR™ with CM exposure as a primary endpoint. The CM exposure in DCR cohort was 36.8 ± 19.2 ml and Control cohort 69.4 ± 27.3 ml without any adverse events (163). Therefore, this approach could be used in patients with CKD as another mean of prevention of CI-AKI. For clear recommendation and implementation of this technique into clinical practice, a randomized trial with hard clinical endpoints is yet required.

## Applicability in non-coronary interventional procedures

Findings from coronary artery procedures translate to structural interventional cardiology with few changes. TAVI has a lower risk of CI-AKI. Despite the higher rate of anemia and the higher degree of CI-AKI in a study comparing TAVI vs. PCI, the occurrence of CI-AKI was significantly lower in the TAVI cohort. (6.7% vs. 14.5%) (164). A recent study calls into question the causative role of CM in CI-AKI in patients undergoing TAVI. Because higher CM volumes did not result in an increase in CI-AKI incidence, the greatest risk comes from pre-procedural low GFR values and procedural factors such as periprocedural hypotension (165, 166). The same can be said for MitraClip interventional procedures. The incidence of CI-AKI was 13%, with pre-procedural risk factors having the greatest influence (167). However, AKI can occur even when no CM is used in certain procedures. The rate of AKI in Swedish patients undergoing CABG was 13% (168).

## Current model of contrast-induced acute kidney injury prevention

**FIGURE 2:**
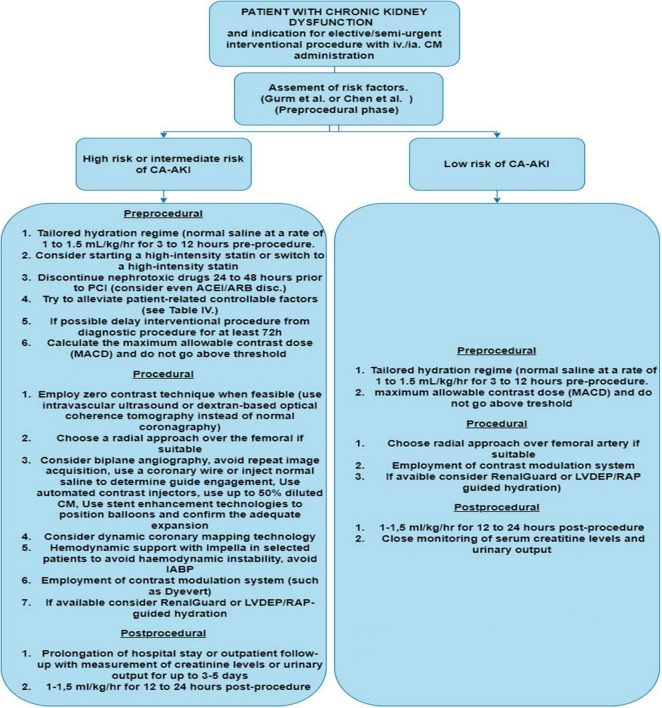
Diagram for Cl-AKI prevention and patient approach.LVDEP, left ventricle end-diastolic pressure; RAP, right atrial pressure; IABP, intra-aortic balloon pump; CM, contrast media; CA-AKI, contrast associated acute kidney injury.

Since the discovery of CA-AKI, prevention recommendations have centerd on the optimizing of hemodynamics and fluid status, avoiding nephrotoxic treatments, and identifying and treating the underlying cause as soon as possible. As a result, the authors of this manuscript suggest the following approach (Figure 2). We recommend using the models proposed by Chen et al. or Gurm et al. for risk prediction due to its external validation and the use of only pre-procedural risk factors. For each patient with advanced CKD, appropriate screening with robust risk model prediction should be performed.

According to the ESC guidelines, every patient with CKD should receive periprocedural hydration with intravenous saline (normal saline at a rate of 1 to 1.5 ml/kg/h for 3–12 h before the procedure and 1–1.5 ml/kg/h for 12–24 h after the procedure). For at least 24 h, all patients should abstain from taking nephrotoxic drugs if feasible (especially NSAIDs, aminoglycosides, and diuretics); preoperative ACEI/ARB discontinuation may be considered if tolerated. Given the low costs and the low risk of side effects, statin therapy should be initiated or titrated to a high-dose statin if already being administered. If the patient has modifiable patient-related risk factors (see Table 6) and coronarography or PCI can be delayed, these risk factors should be corrected and, if possible, staged PCI performed.

If a patient is classified as high to intermediate risk of CI-AKI, it should be proceeded with all contrast-reducing and non-contrast procedures accessible. If available, the use of Renal Guard or a comparable approach for tailored aggressive intravenous fluid therapy (such as LVEDP, right atrial pressure or BIVA guided hydration) should be considered. Based on the findings of meta-analyses and trials with the DyeVertTM system, the use of such a device may be useful in any CKD patient undergoing an interventional or diagnostic procedure with CM. If available, techniques for dynamic coronary vessel imaging, biplane imaging and stent visualization (such as ClearStent™ by Siemens etc.) should be used. If technically feasible, new biomarkers for early diagnosis and prediction of CI-AKI could be used (namely Cystatin C, combination of [TIMP-2] × [IGFBP7]). This method could aid in an earlier CI-AKI diagnosis and eventual intervention.

## Directions for the future management and prevention

We propose developing a new risk prediction model based on standard risk factors of CI-AKI yet including novel diagnostic possibilities such as inflammatory hematological and biochemical parameters. The inflammatory process in the kidney is complex, but in the early stages of CI-AKI, neutrophils are recruited first, followed by lymphocytes. As a result, we could include in risk prediction models the neutrophil-to-lymphocyte ratio (NLR), segmented-neutrophil-to-monocyte ratio (SMR), neutrophil-lymphocyte-platelet ratio (NLPR), red blood cell distribution width, and hsCRP. These values are usually known ahead of time or can be easily calculated with no added costs. Because of ongoing research with novel CI-AKI biomarkers such as NGAL, TIMP, IGLF 17, and microRNAs, future early prediction models may rely on them.

The treatment optimization aimed at recognizing and correcting identified risk factors may reduce the likelihood of CA-AKI. Thus, the treatment customization based on biochemical and hematological values measured should be critical for the future management of CI-AKI. Recent CI-AKI research focuses on developing a potent antioxidant or vasoactive agent capable of reducing vasospasm or the intrinsically cytotoxic properties of CM. Recent studies with nicorandil, trimetazidine, inorganic nitrate, or pentoxifylline have produced promising results, but these should be confirmed by a robust multicenter study yet. Recent trials with antioxidant inducers such as Heme-oxygenase may be a future prevention method, but definitive evidence of its benefit is still required. Remote ischemic preconditioning and protein isolation responsible for its beneficial effect could have a significant impact not only on CI-AKI prevention but also on AKI prevention in general.

The most significant technological advances in the prevention of CI-AKI occurred in alternative imaging technologies, contrast modulation systems and interventional procedures. Assuming the future decrease of the costs and better availability of these methods, their general applicability in most situations could significantly reduce the CI-AKI prevalence. As a result, completely zero contrast, non-iodinated CM, or ultra-low contrast techniques may be routinely used in high risk patients in the future.

## Conclusion

Contrast-induced acute kidney injury represents a clinically significant complication. Its importance has increased because of the increasing number different iodinate CM investigations. The multitude of variable confounding factors and frequent exclusions of patients with a substantial CI-AKI risk have not allowed the creation of universal rules for patients undergoing imaging procedures with CM administration. In addition to the recommended standard fluid hydration protocol and to the CM reduction and modulation, some novel approaches have been studied involving individualized hydration and sophisticated tools minimizing the CM administration. Specifically, we discuss novel approaches advocating for zero contrast, ultra-low or alternative media administration. The general applicability of these approaches proved to be safe and feasible yet limited by the top-tier availability. Another promising method such as carbon dioxide angiography are discussed: based on current animal studies, this method could be extended to be used above diaphragm angiographies without any safety concerns, if future clinical trials prove this. We also discuss advanced dynamic coronary mapping technology, which aids in orientation during low to zero contrast interventional cardiology procedures. Understanding of pathogenesis and advances in proteomics allowed us to locate the specific molecule responsible for cell death and the initiation of pro-apoptic pathways in injured renal cells. As a result, the new age of targeted therapy and novel biomarkers appears to be on the horizon. Finally, we discussed the implications of the RIPC findings, which, despite being a simple preventive method, has no adverse effects and could be offered to the patients with a higher risk of CI-AKI. There is, however, an unmet need for additional multicenter randomized trials with large cohorts to clarify effect of discussed prevention methods on CI-AKI incidence reduction. Such trials should ideally include longer follow-up periods and should also study non-cardiovascular procedures. Patients with severe chronic renal insufficiency and those on hemodialysis also need to be investigated.

## Author contributions

All authors listed have made a substantial, direct, and intellectual contribution to the work, and approved it for publication.
